# En bloc discectomy via anterior lumbar approach: a technical note

**DOI:** 10.1051/sicotj/2026002

**Published:** 2026-04-20

**Authors:** Harmantya Mahadhipta, Amri Muhyi, Muhamad Abdul Harist Fadhlizain, Mitchel Mitchel, Karina Sylvana Gani, Erica Kholinne

**Affiliations:** 1 Department of Orthopaedics and Traumatology, Tangerang General Hospital Banten 15117 Indonesia; 2 Department of Orthopaedics and Traumatology, Cipto Mangunkusumo Hospital, Faculty of Medicine, Universitas Indonesia Jakarta 10430 Indonesia; 3 Orthopedics and Traumatology, Gatam Institute, Eka Hospital Tangerang 15321 Indonesia; 4 General Practitioner of Tangerang General Hospital Banten 15117 Indonesia; 5 Department of Surgery, Faculty of Medicine, Universitas Trisakti Jakarta 11440 Indonesia

**Keywords:** Lumbar vertebrae, Intervertebral disc displacement, Discectomy, Total disc replacement, Implant subsidence

## Abstract

*Introduction:* Implant subsidence remains one of the complications following lumbar interbody fusion and total lumbar disc replacement, often attributed to excessive and uneven preparation of the subchondral bone. To address this limitation, we describe a novel surgical approach – en bloc discectomy – designed to enable more controlled disc removal, preserve subchondral endplate integrity, and minimize the risk of implant subsidence. *Methods:* We describe the procedural steps for the en bloc discectomy, including patient positioning, surgical approach, and the specific technique using a Cobb spinal elevator to remove the cartilaginous en bloc. The technique’s advantages include controlled disc removal, minimized subsidence, and even subchondral endplate preparation. *Results:* En bloc discectomy was successfully performed in our patient. No intraoperative or postoperative complications occurred, and all patients reported immediate and sustained symptomatic improvement. *Conclusion*: En bloc discectomy provides a safe and reproducible alternative to conventional (standard piecemeal discectomy) disc excision. By reducing endplate damage and implant subsidence, this technique has the potential to improve long-term stability and clinical outcomes in patients undergoing lumbar interbody procedures.

## Introduction

Lumbar disc herniation and degenerative disc disease are major causes of low back pain and radiculopathy, often requiring surgery when conservative treatment fails [1]. Anterior lumbar interbody fusion (ALIF) is commonly performed because it provides direct disc access and restores disc height and alignment with limited posterior disruption [2]. However, implant subsidence, sinking of the interbody device into the endplate, remains a significant complication. Lumbar Disc Replacement (LDR) offers better stabilization and has been associated with lower subsidence rates than ALIF [3]. Reported subsidence rates for ALIF range from 6% to 23%, whereas Richard et al. documented a 3.4% rate for LDR. Subsidence may compromise fusion stability, reduce lordosis, cause recurrent symptoms, and necessitate revision surgery [4].

Successful lumbar interbody implantation via the anterior approach requires balancing two opposing goals: removing disc material to prepare a fusion bed while preserving endplate integrity for load-bearing. Conventional piecemeal discectomy can leave residual cartilage, is difficult to standardize, and risks damaging the subchondral bone [1, 2]. Excessive endplate removal weakens axial strength; one biomechanical study showed failure load reductions of 15% in TLIF and 36.6% in PLIF after aggressive preparation [5]. In ALIF, subsidence has been associated with over-preparation, cage–endplate mismatch, and suboptimal cage positioning [6], yet the ideal extent of endplate removal remains unclear. Similarly, size mismatch in lumbar disc replacement can cause the implant to rest on weaker central bone rather than the stronger peripheral ring, increasing subsidence risk [7]. Therefore, a more reproducible and endplate-preserving disc removal technique is needed.

To address these challenges, we introduce a novel en bloc discectomy technique for the anterior lumbar approach. Using a Cobb spinal elevator, this method enables controlled, systematic removal of the cartilaginous disc while minimizing endplate trauma. This technical note outlines the key procedural steps and presents illustrative clinical cases. Our goal is to provide a reproducible technique that achieves adequate disc clearance and may help reduce implant subsidence in anterior lumbar interbody procedures.

## Surgical technique

Standard surgical instruments were utilized, including a Cobb elevator for disc space preparation, electrocautery for soft tissue management, and forceps for tissue handling. A third-generation total disc replacement implant, ProDisc^®^-L (Synthes Spine, West Chester, PA), was implanted following discectomy and endplate preparation, with attention to appropriate sizing and positioning based on preoperative planning.

## Anesthesia, patient position, and incision

**Figure 1:**
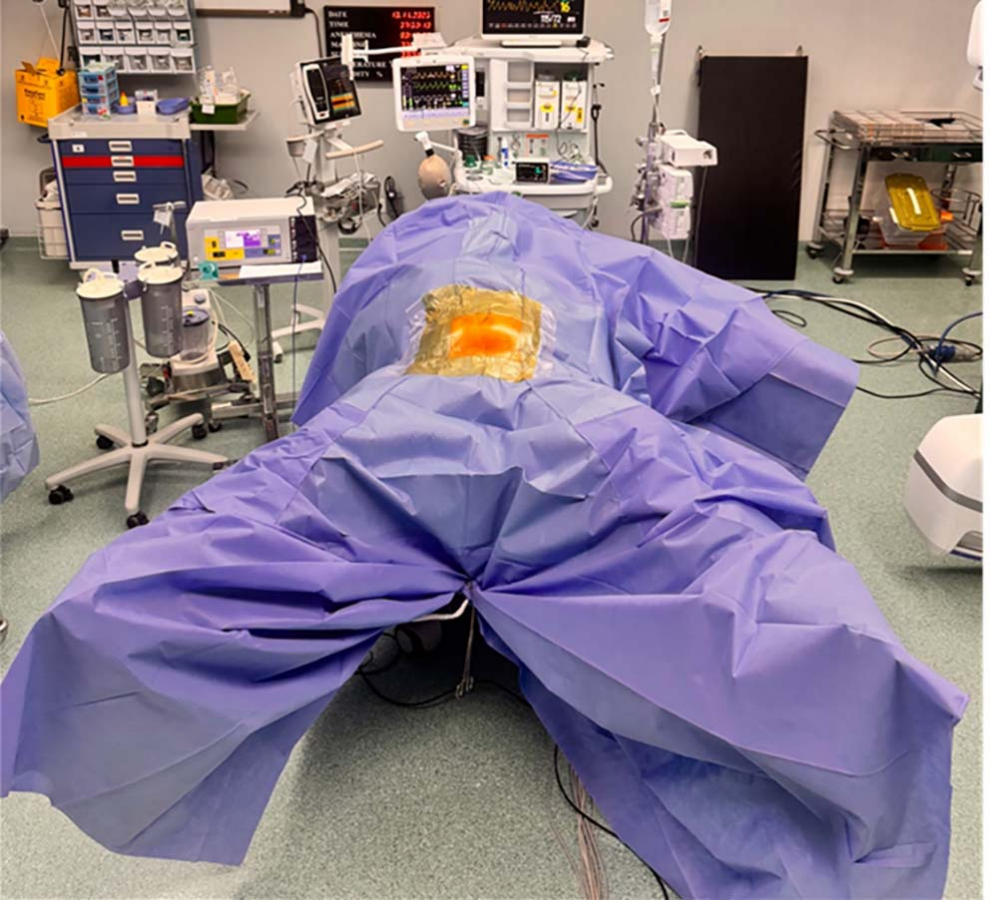
Patient was positioned in supine Da Vinci under sterile operative procedure.

All procedures were performed under general anesthesia. The patient was positioned supine in the Da Vinci position on the operating table and prepared in a standard sterile fashion (Figure 1). The surgeon stood on the caudal side of the patient. The surgical exposure is achieved through a midline or paramedian incision at all lumbar levels, or a mini-Pfannenstiel incision at the L5/S1 level, utilizing a retroperitoneal approach with vascular mobilization and dissection. This approach offers several advantages, including direct midline visualization of the disc space and broad lateral exposure of the vertebral bodies, enabling efficient disc removal and rapid endplate preparation [8]. In addition, anterior access permits the use of larger interbody implants with increased surface area, facilitating more effective correction of spinal pathology. Preservation of the posterior spinal musculature and anterolateral psoas muscles may also contribute to reduced postoperative pain and functional impairment [9]. We then cauterized the anterior longitudinal ligament and the anterior portion of the disc, creating a rectangular window. Intraoperative fluoroscopy was used to confirm the target disc level.

## Identification of disc–endplate borders

**Figure 2:**
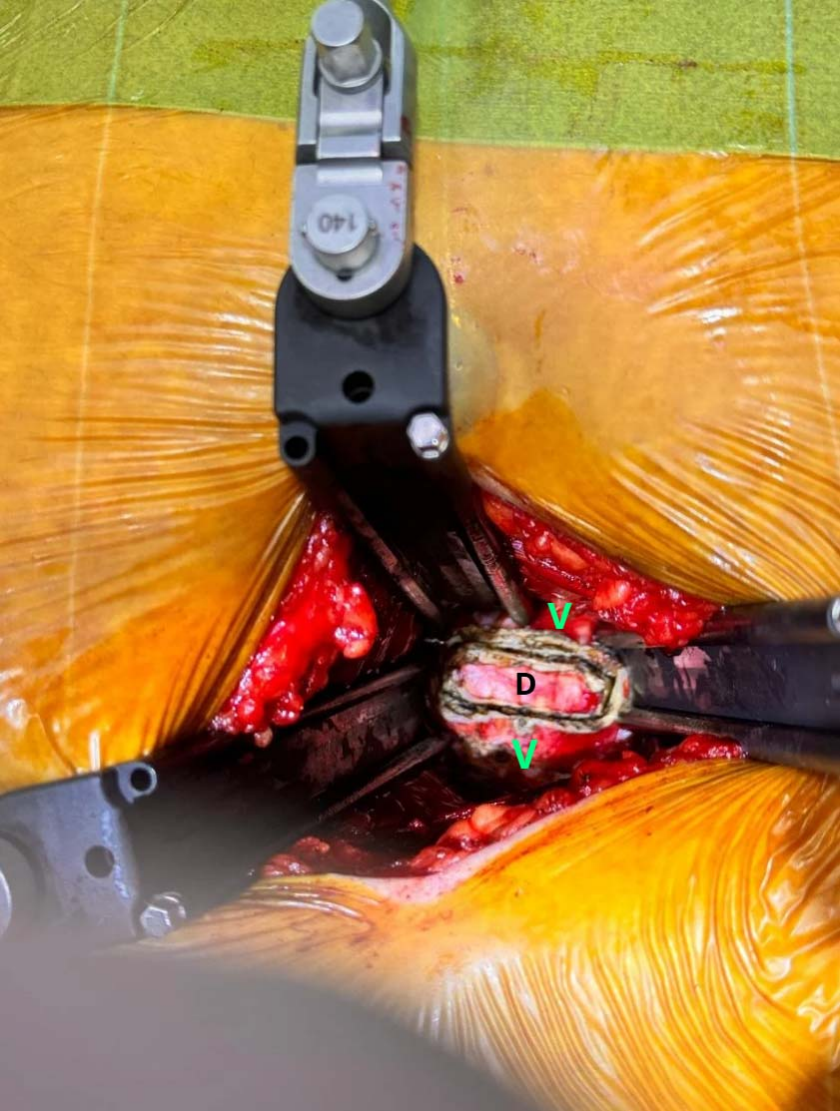
The anterior part of the targeted disc was cauterized into a rectangular shape. “V” represent as vertebral body, and “D” represent as disc.

Following exposure, the anterior annulus fibrosus was incised longitudinally with an electrocautery/diathermy. The incision was made large enough to allow adequate visualization and working access while preserving the peripheral annular fibers to maintain stability. The anatomical borders of the working zone were then carefully identified, the superior and inferior limits were defined at the annulus-endplate junction, while the lateral borders were established approximately 1cm medial to the lateral edge of the disc, corresponding to the annulus-nucleus junction (Figure 2).

**Figure 3:**
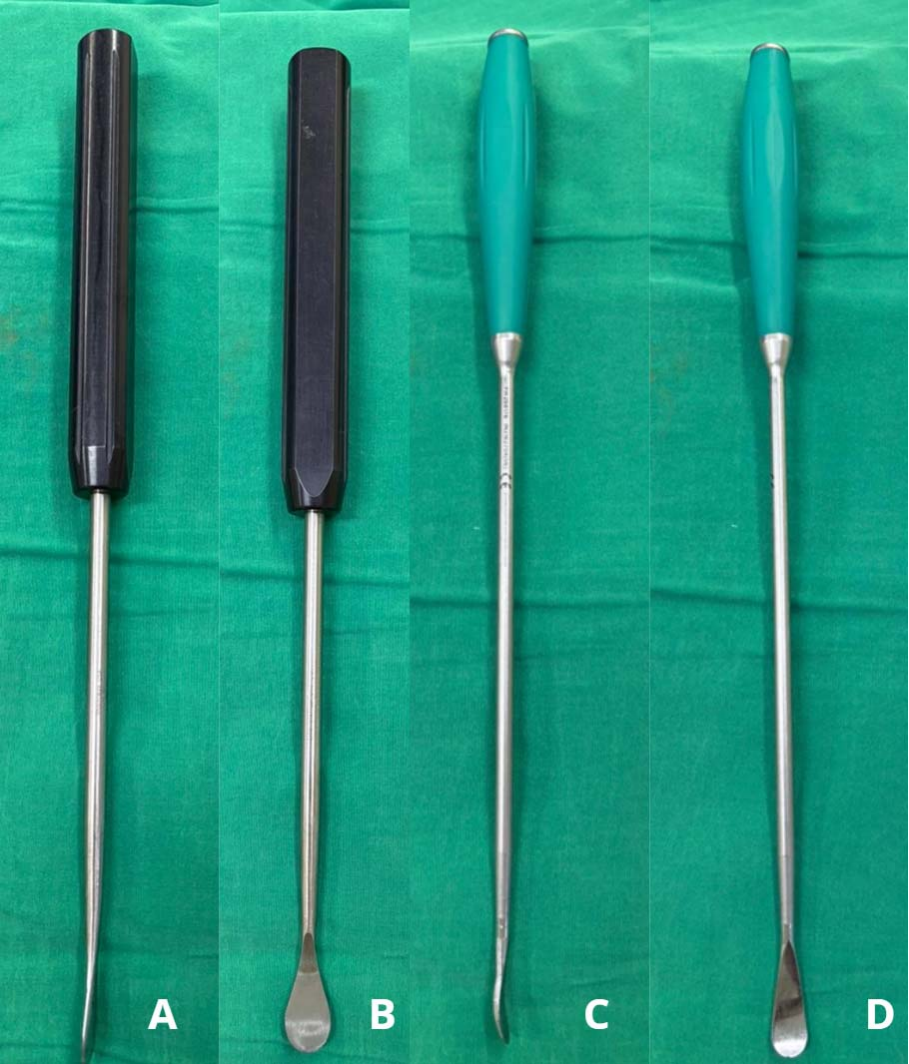
Cobb elevator (A&B) flat head (C&D) curve head.

**Figure 4:**
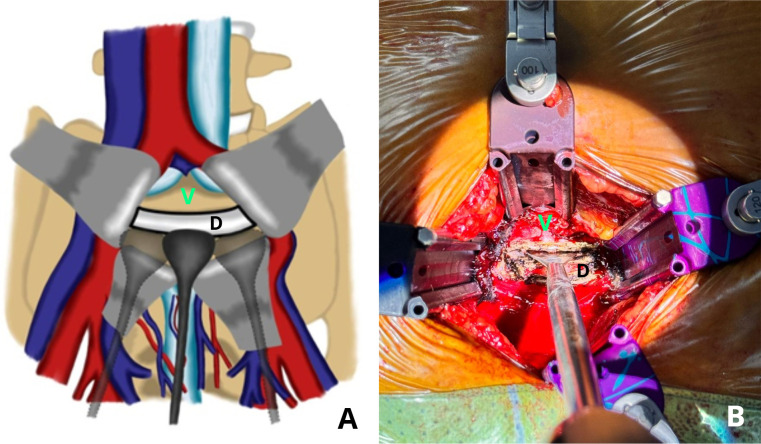
(A) Illustrative images. (B) Intraoperative images showing detachment of the superior and inferior parts of the nucleus pulposus from the posterior annulus using a flat head Cobb elevator, by “sweep and lift” technique. “V” represent as vertebral body, and “D” represent as disc.

**Figure 5:**
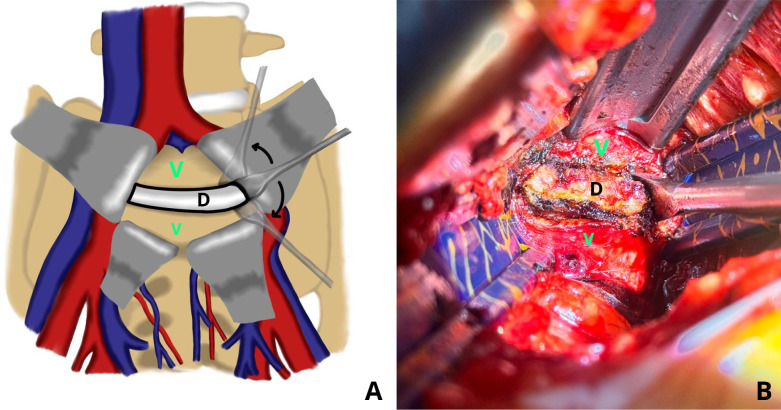
(A) Illustrative image. (B) Intraoperative images of the lateral detachment from the lateral annulus using a curved head Cobb elevator, by the “scooping” technique. “V” represent as vertebral body, and “D” represent as disc.

Dissection planes were then carefully developed superiorly and inferiorly between the vertebral endplates and the intervertebral disc. We are using two types of Cobb elevators: a flat-head for the superior and inferior portions, and a curved head for the lateral portion of the nucleus (Figure 3). Using a flat-head Cobb elevator, the cartilaginous endplate was elevated from the subchondral bone by the “sweep and lift technique” from the superior and inferior cartilage endplate. The lateral portion of the nucleus pulposus was detached from the lateral annulus using a curved head Cobb elevator by the “scooping technique” (Figures 4 and 5). Removal of the disc en bloc, which consists of the anterior annulus and the whole nucleus pulposus.

## En bloc discectomy

**Figure 6:**
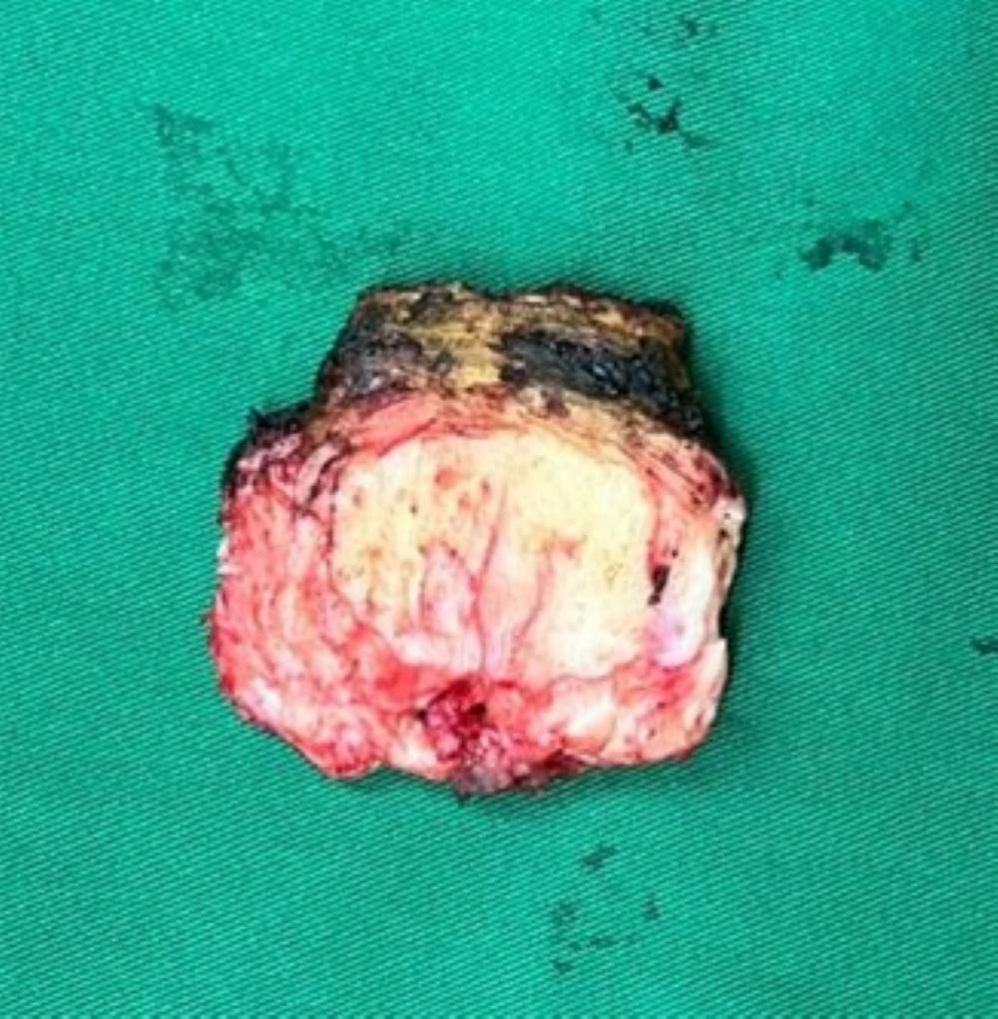
The excised disc fragment.

The posterior annulus was removed using a Kerrison punch to ensure canal decompression. Disc material was removed centrally while carefully preserving the peripheral subchondral bone and ring apophysis to maintain load-bearing capacity and reduce the risk of cage subsidence. Any residual fragments were cleared with pituitary rongeurs or curettes as needed. The excised disc (Figure 6) was measured to guide cage sizing. Sequential trial implants were then inserted to assess disc height restoration, footprint coverage, and sagittal alignment, with care taken to avoid over-distraction and potential endplate injury.

**Figure 7:**
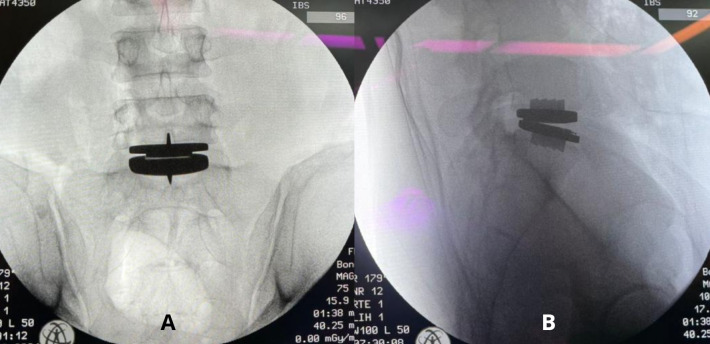
The interbody cage is positioned at the L5-S1 level as seen under fluoroscopic imaging.

The appropriately sized interbody device was inserted into the prepared disc space under fluoroscopic guidance. The device was positioned centrally to maximize contact with the peripheral ring apophysis, thereby optimizing axial load distribution and construct stability. Final placement and alignment were confirmed with anteroposterior and lateral fluoroscopic imaging (Figure 7).

After meticulous hemostasis, retractors were released, and the retroperitoneal contents were allowed to return to their anatomical position. A layered closure was performed, beginning with the fascia, followed by the subcutaneous tissue and skin. We have summarized a key surgical step and potential pitfalls in En Bloc Discectomy via Anterior Lumbar Approach (Table 1).

**Table 1:** Pearls and pitfalls of en bloc discectomy via anterior lumbar approach.

**Pearls**	**Details**
Direct visualization of the disc and anterior pathology	The anterior approach provides direct access to both the disc space and any anterior intracanal pathology, facilitating better surgical precision
Complete nucleus removal	Complete removal of the nucleus offers a reduced risk of re-herniation compared to traditional microdiscectomy, ensuring long-term relief
Preserving subchondral bone with Cobb elevators	By positioning Cobb elevators parallel to the endplate, the attachment of the cartilage endplate can be carefully removed, preserving the critical subchondral bone for optimal bony integration with the implant.
Stability through peripheral annulus preservation	Preserving the peripheral annular rim (especially on the lateral side) enhances implant stability, minimizing the risk of displacement.
Larger implant placement for increased stability	The anterior approach allows for the placement of a larger cage or implant, contributing to better overall stability and reduced risk of implant migration
**Pitfalls**	
Steep learning curve for anterior approach	The anterior lumbar spine approach requires a steep learning curve, as it necessitates familiarity with the vascular structures in the area to avoid complications.
Risk of subchondral bone damage	Use of aggressive, non-parallel Cobb elevators may compromise the integrity of the subchondral bone, increasing the likelihood of implant subsidence or failure.
Implant misalignment and malposition	Incorrect alignment or positioning of the implant can lead to instability and may increase the likelihood of requiring revision surgery due to mechanical failure

## Potential complication and risks

En bloc resection is a surgical method designed to excise pathological tissue in one intact piece to obtain clear margins and minimize the likelihood of residual disease. However, despite its oncologic and biomechanical benefits, this technique is technically challenging and carries a greater risk of complications than piecemeal resection [10, 11]. En bloc resection necessitates the removal not only of the involved bone but also of most of the surrounding stabilizing structures, resulting in complete spinal instability [12]. With the en bloc discectomy approach, common intraoperative complications include dural tears that will lead to cerebrospinal fluid (CSF) leakage, visceral and vascular injuries, malpositioning of surgical hardware, and nerve root injury, causing a longer hospitalization period [12–14]. Epidural bleeding is also a potential concern [12]. A retrospective study found that the most frequent intraoperative complication was dural tear, occurring in approximately 34% of cases, while the most common postoperative complication was hematoma, with an incidence of about 12%. In the late postoperative period, there is a substantial risk of construct failure, with or without loss of correction. Such events are relatively common in spinal surgery because of the complex biomechanics and kinematics of the spine [15].

## Discussion

Lumbar fusion is the most common surgical option for discogenic pain or spinal instability unresponsive to conservative treatment [16]. Among spinal fusion techniques, the anterior approach is widely utilized because of its high success rates and relatively low complication profile, and is indicated for selected degenerative lumbar disorders, including discogenic or facet-mediated low back pain, neurogenic claudication, radiculopathy due to foraminal stenosis, and degenerative spinal deformities such as symptomatic spondylolisthesis and degenerative scoliosis. This approach is best suited for the L4/L5 and L5/S1 levels, where anterior access is generally safer and facilitates restoration of disc height and sagittal alignment. In contrast, its application at the L2/3 and L3/4 levels is limited due to the need for extensive peritoneal and renal mobilization and the rare but potential risk of superior mesenteric artery thrombosis. Contraindications to ALIF include extensive prior abdominal surgery with adhesions, unfavorable vascular anatomy, severe peripheral vascular disease, a solitary kidney on the side of exposure, active spinal infection, and high-grade (Grade ≥2) degenerative spondylolisthesis in the absence of posterior instrumentation. [9, 17]. One notable complication is postoperative subsidence, defined as a reduction in disc height [18]. To avoid fusion-related drawbacks, lumbar total disc replacement (TDR) was introduced. TDR achieves pain relief through complete disc removal, restoration of physiological load transmission and sagittal alignment, preservation of motion, and may reduce adjacent-segment pathology [19]. However, TDR has also been associated with drawbacks, such as subsidence, dislocation, or malposition of the implant [20]. However, the anatomical structure of the vertebral body also influences the risk of subsidence. The peripheral endplate, or epiphyseal ring, is mechanically stronger than the central region. Therefore, implants with a larger footprint are preferred to distribute load across these stronger areas and reduce focal stress. By engaging the epiphyseal rings more effectively and occasionally interacting with pedicle screws as a mechanical stop, larger implants are associated with a lower incidence and severity of subsidence [21]. The clinical improvement and subsidence rates in different techniques are summarized in Table 2.

**Table 2:** Comparison of clinical outcomes and subsidence rates at final follow-up among various surgical techniques.

Author (Year)	Total of patients *n* (M/F)	Age (years ± SD)	Surgical technique	Follow-up (months)	Implant type	Subsidence rate (%)	Clinical outcome (mean ± SD)
Cuellar et al (2021) [22]	46 (N/A)	N/A	ALIF	72	ProDisc-L (Synthes Spine, Westchester, PA)	N/A	Improved ROM
							ODI
							TDR-1: 46% improvement
							TDR-2: 40% improvement
							TDR-3: 38.6% improvement
							VAS
							TDR-1: 53%
							improvement
							TDR-2: 58% improvement
							TDR-3: 55% improvement
Rao et al (2017) [23]	147 (103/44)	57.3 ± 13.6	ALIF	18	SynFix-LR PEEK integral (Depuy) for 89.1% of the patients	10.2%	VAS: 2.7 ± 0.2
							ODI: 28.8 ± 1.8
							SF-12 physical: 41.7 ± 0.9
							SF-12 mental: 48.9 ± 1.0
							Fusion rate: 91.2%
Chen et al (2019) [24]	107 (53/54)	60.79 ± 1.12	LLIF	24	PPEK cages	26.9%	VAS: 2.40 ± 0.32
							JOA: 18.67 ± 0.46
Gionali et al (2024) [25]	61(38/23)	39 ± 13.3	LLIF	35.5	Static (NuVasive CoRoent ® or Modulus ®), Static (J&J Synmesh ®), Expandable (Globus Medical ELSA ®)	27.8%	MacNab criteria:
							Excellent: 65.6%
							Good: 29.5%
							Fair: 4.9%
							Poor: 0
Chang et al (2019) [26]	169 (61/108)	67.7 10.9	OLIF	12	PEEK cages	62/168 (36.9%) 85/261 (32.6%)	ODI
							DS: 37.7 ± 13.5
							SS: 34.4 ± 5.1
							ST: 38.2 ± 15.2
							DF: 55.0 ± 14.9
							VAS
							DS: 2.4 ± 1.9
							SS: 2.2 ± 1.6
							ST: 2.0 ± 1.6
							DF: 3.8 ± 1.6
							SF-36
							DS: 38.6 ± 7.6
							SS: 39.0 ± 5.8
							ST: 39.6 ± 7.4
							DF: 30.9 ± 6.6
							JOABPEQ
							All domains improved
Wen et al (2020) [27]	74 (28/46)	BPS: 56.9 ± 13.2	OLIF	24 months	N/A	13/74 (17.6%)	VAS
		UPS: 58.9 ± 16.1					BPS: 2.97 ± 0.45
							UPS: 2.73 ± 0.83
							ODI
							BPS: 8.17±1.74
							UPS: 7.94±1.62
Oh et al (2017) [28]	129 (52/87)	65.17 ± 8.599	PLIF	49.2	PEEK cage (O.I.C. cages; Stryker	15.8%	VAS: 2.89
							ODI: 15.86
							SF-36: 16.46
Park et al (2019) [29]	40 (11/29)	68.9 ± 7.9	PLIF	29.7	N/A	Low grade; 10/44 (22.7%)	VAS:5.2 ± 2.1
							ODI: 51.9 ± 16.1
						High grade; 4/44 (9.1%)	
Zhao et al (2020) [30]	76 (29/47)	53.91 ± 1.13	TLIF	60	PEEK material and cuboid shape (arched appearance) from Stry	Low grade: 11.8%	VAS:2.21 ± 0.05
							ODI: 10.34 ± 1.03
						High grade: 7.9%	
							JOA: 20.79 ± 0.40
Kulkani et al (2024) [31]	36 (N/A)	42.16 ± 19.7	TLIF	48	N/A	N/A	VAS
							Group A: 0.19
							Group B: 0.29
							ODI
							Group A: 5
							Group B: 5

Abbreviations: N/A, Non Available; M, male; F, female; ALIF, anterior lumbar interbody fusion; DF, deformity; DS, degenerative spondylolisthesis; SS, spondylolytic spondylolisthesis; ST, spinal stenosis; ROM, range of motion; BPS, bilateral pedicle screw; UPS, unilateral pedicle screw; LLIF, lateral lumbar interbody fusion; OLIF, oblique lumbar interbody fusion; PEEK, polyetheretherketone; PLIF, posterior lumbar interbody fusion; TDR-1, Total Disc Replacement 1 level; TDR-2, Total Disc Replacement 2 level; TDR-3, Total Disc Replacement 3 level; SF- 12, 12- Item Short Form Health Survey; TLIF, transverse lumbar interbody fusion; VAS, visual analog scale; SF- 36, 36- Item Short Form Health Survey; ODI, Oswestry Disability Index; JOABPEQ, Japan Orthopedic Association Back Pain Evaluation Questionnaire; JOA, Japan Orthopedic Association.

Preserving cartilaginous endplates is critical in lumbar fusion, as endplate violation significantly increases subsidence risk. Subsidence can lead to unintended fusion or TDR failure due to wear or implant displacement [32]. Kitzen et al. [16] reported parallel subsidence in 6.4% of patients. The en bloc discectomy technique offers a controlled method to remove the posterior disc, often the source of neural compression, while protecting the endplate. We applied this technique in three patients, all without complications.

Cage shape, size, and placement all affect subsidence risk. Larger-footprint ALIF cages reduce subsidence by distributing load more evenly across the endplate. Implant placement along the vertebral periphery is preferred because the central endplate is structurally weaker [4]; cadaveric studies confirm that peripheral positioning helps prevent cage penetration regardless of design [33]. Taller cages increase subsidence risk due to over-distraction and higher endplate loading, whereas undersized cages may compromise decompression and fusion success [4].

Based on our earlier experience with conventional discectomy techniques, several technical limitations were consistently encountered. Disc removal performed in a piecemeal fashion often resulted in incomplete clearance of disc material, leading to concerns regarding the cleanliness of the disc space. Manual curettage frequently produced uneven endplate surfaces, with focal areas of excessive excavation adjacent to inadequately prepared regions. This nonuniform endplate preparation may compromise implant support and potentially increase the risk of subsidence.

In addition, the procedure required a longer operative time, particularly during endplate preparation using curettes. These accumulated technical challenges informed the transition to an en bloc discectomy technique, which aligns with the advantages observed in the present study. Based on our study and experience, en bloc discectomy provides a more controlled and systematic method of disc removal, thereby reducing procedural complexity and operative time. By enabling disc clearance in a single piece, this technique minimizes the likelihood of residual disc fragments while ensuring adequate decompression. Importantly, the focused removal of the cartilaginous disc en bloc reduces the need for aggressive curettage, facilitating more uniform endplate preparation and preserving vertebral endplate integrity. This standardized and reproducible approach allows for improved intraoperative assessment of endplate quality, which may enhance implant seating and contribute to a lower risk of implant subsidence.

The limitations of this technique include a significant learning curve, a heightened risk of endplate injury, and the possibility of incomplete discectomy. Because it requires a high level of precision, improper use of an osteotome or specialized curette may result in endplate fracture or damage. Furthermore, incomplete removal of the disc block can leave residual fragments, increasing the risk of re-herniation or persistent neural compression.

## Conclusion

The en bloc discectomy is a promising technique for improving the outcomes of anterior lumbar fusion by focusing on precise and complete disc removal while preserving the endplates. This approach addresses the limitations of traditional piecemeal discectomy and aims to provide better long-term stability and reduce complications.

## Data Availability

This article has no associated data generated and/or analyzed.
